# From Habit to Justification: A Dual Process Theory Approach to Primary Care Bypassing in China

**DOI:** 10.1007/s10728-025-00548-9

**Published:** 2025-11-04

**Authors:** Bo Li

**Affiliations:** Department of Applied Social Sciences, The Hong Kong Polytechnic University, Hong Kong, China

**Keywords:** Tiered healthcare system, Primary care utilisation, Patient decision-making, Dual process theory, Health-seeking behaviour

## Abstract

China’s tiered healthcare system is designed to guide patients through a two-way referral process, with primary care as the first point of contact. However, prevalent bypassing of lower-tier facilities undermines the system’s effectiveness. While previous research has examined healthcare choices, the cognitive drivers of bypassing behaviour remain insufficiently understood. A case study design was employed, involving 23 semi-structured interviews with hypertensive patients in Shenzhen. Deductive thematic analysis, guided by dual process theory, explored the cognitive mechanisms underlying bypassing, focusing on intuitive (System 1) and deliberative (System 2) decision-making. Bypassing behaviour emerged from the interplay between Systems 1 and 2 processes. System 1, driven by automatic heuristics, reinforced habitual hospital use and social norms, maintaining status quo bias and the belief that tertiary hospitals offer superior care. System 2, involving reflective risk assessment, led patients to favour hospitals due to concerns over service quality and adequacy. Importantly, System 1 established bypassing as the default, while System 2 provided retrospective justifications, entrenching this decision. However, a subset of patients, influenced by health status and personal circumstances, exhibited more flexibility, opting for primary care for convenience or accessibility. This delineates the cognitive model’s boundary conditions, revealing that behavioural outcomes are not deterministic but contingent on individual and situational factors. The application of dual process theory offers a nuanced understanding of bypassing behaviour within China’s tiered healthcare system, emphasising the roles of both instinctive and analytical cognitive processes. Findings suggest the need for multidimensional interventions targeting both cognitive pathways to improve primary care utilisation.

## Introduction

A tiered healthcare system structures care delivery across primary, secondary, and tertiary levels based on patient needs. Its goal is to optimise resource allocation by directing routine, preventive, and chronic care to primary services while reserving higher-tier facilities for complex treatments. This system is designed to reduce pressure on specialised hospitals, enhance community-based care, and improve efficiency and access [15]. However, its effectiveness largely depends on patient adherence to referral pathways and the ability of primary care to meet diverse healthcare needs—challenges that are particularly pronounced in China [10, 13, 16, 17].

China’s tiered healthcare system was introduced to address inefficiencies in healthcare utilisation. Historically, patients sought care primarily at large urban hospitals, even for minor ailments [6]. This overreliance on tertiary facilities led to overcrowding, inefficient resource allocation, and widening disparities in service availability [13, 16, 17]. In response, the government launched a series of reforms, including the Healthy China 2030 initiative, to strengthen primary care and establish a structured two-way referral system [12]. These reforms sought to channel patients towards community health centres for routine care while reserving hospitals for specialised treatments [17]. However, despite these efforts, many patients continue to bypass primary care in favour of higher-tier hospitals [29, 31, 34, 36, 37], sustaining congestion in tertiary facilities and undermining the system’s intended efficiency.

Literature identifies several factors driving this bypassing behaviour. Structural barriers remain a key limitation, as many patients perceive primary care as inadequate or inaccessible [10, 31, 34, 37]. Community health centres often lack skilled personnel, advanced medical technology, and strong integration with higher-tier hospitals [33, 34], diminishing their appeal. This resource disparity is partly systemic; historical underinvestment and a focus on hospital-centric development have created a feedback loop where talented practitioners are drawn to better-equipped, higher-status hospitals [11], further limiting the scope and sophistication of services that primary care can offer. The referral system also remains fragmented [17], further discouraging primary care utilisation. Without robust, formalised pathways and shared electronic health records, primary care providers function as isolated gatekeepers rather than care coordinators [10]. This lack of integration limits their ability to manage patients effectively across tiers and reinforces their perceived role as merely a steppingstone to ‘real’ care in hospitals.

Patient perceptions and trust in primary care significantly influence healthcare choices [37]. Many patients believe tertiary hospitals provide superior care [16, 34], regardless of their condition’s complexity. Concerns over primary care providers’ competence [32–34], particularly in managing complex conditions, reinforce this perception. These concerns are often rooted in a reality where primary care physicians, facing high patient volumes with limited support, have fewer opportunities for professional specialisation or continuous education in chronic disease management compared to their hospital-based counterparts [12]. Additionally, the lack of continuity in care, where patients frequently see different providers, further erodes trust and leads them to seek care at hospitals where they feel more familiar with medical staff and treatment processes [36].

Sociocultural norms also shape bypassing behaviour. In urban areas, tertiary hospitals are widely perceived as symbols of medical excellence [36], reinforcing a cultural preference for specialist care. Social networks, family advice, and peer influence further encourage patients to seek treatment at higher-tier facilities [14], even when primary care is appropriate. Behavioural biases, such as status quo bias (patients default to familiar healthcare settings), further entrench these patterns [16].

Economic factors play an additional role. Within China’s fee-for-service and diagnosis-related group reimbursement models, primary care providers receive lower reimbursement rates for consultations and procedures than specialists in tertiary hospitals [11]. This creates a financial disincentive for primary care facilities to expand services or for practitioners to specialise in chronic disease management, as it is less financially rewarding [19, 30]. Consequently, this can perpetuate perceptions of a lower quality of care [34, 37]. Meanwhile, patients often accept the higher costs of hospital-based care, perceiving it as more reliable and specialised [25].

While these perspectives provide valuable insights, much of the literature adopts a one-dimensional approach, overlooking cognitive and behavioural mechanisms that shape healthcare decision-making. Structural barriers are often framed in terms of resource limitations [10], yet this neglects the automatic decision-making processes that guide patient choices. Cognitive shortcuts, such as habitual behaviours or reliance on familiar options [21], play a significant yet underexplored role in bypassing primary care.

Similarly, research on trust in primary care often fails to account for cognitive biases, such as status quo bias or the availability heuristic, which influence how trust is developed and maintained [38]. These biases contribute to the persistent preference for higher-tier hospitals, even when primary care may offer equally or more appropriate care.

Sociocultural influences are typically examined through cultural norms and specialist care preferences [7], yet the cognitive foundations of these behaviours remain underexplored. The tendency to seek specialist care may not solely reflect cultural attitudes but also deep-seated cognitive biases that prioritise familiarity and perceived superiority. Likewise, economic barriers are often studied in isolation, despite the potential for cognitive framing to influence how patients mentally process financial constraints when making healthcare decisions [18].

This study advances understanding of patient bypassing behaviour in China’s tiered healthcare system by examining the cognitive mechanisms that drive healthcare choices. Grounded in dual process theory, it investigates how both intuitive and deliberative cognitive processes shape patients’ tendencies to bypass primary care in favour of higher-tier hospitals. By elucidating the interplay between automatic decision-making and reflective risk assessments, this study provides new insights into the persistent underutilisation of primary care despite extensive structural reforms. Findings contribute to primary care utilisation research by integrating cognitive theory and offer implications for policy and clinical practice.

## Methods

### Theoretical Framework

Dual process theory provides a framework for understanding decision-making by distinguishing between two cognitive systems: an intuitive, automatic system (System 1) and a deliberative, analytical system (System 2) [3]. This theory posits that decisions are shaped by both fast, heuristic-driven judgements and slower, effortful reasoning. System 1 operates rapidly, drawing on heuristics—mental shortcuts that simplify complex decision-making under uncertainty—as well as prior experiences and implicit biases, while System 2 engages in deliberate evaluation, weighing risks and alternatives before reaching a conclusion [4].

Heuristics are a central component of System 1 processing. Within the context of health behaviour, these automatic cognitive shortcuts are influential, as they enable individuals to navigate affectively charged and complex decisions, such as selecting a healthcare provider, without engaging in effortful deliberation each time. This study focuses on three heuristics particularly pertinent to bypassing behaviour: status quo bias (a preference for maintaining a current behaviour or default option), availability heuristic (reliance on immediate examples or anecdotes that come to mind when evaluating a topic), and social norms (the influence of perceived behavioural patterns of one’s peers, leading to conformity).

Applied to healthcare decision-making, dual process theory suggests that patient choices, including the decision to bypass primary care, result from the interaction of these two systems [20]. System 1 fosters habitual health-seeking behaviours shaped by past experiences, social norms, and cognitive shortcuts [5, 24]. For instance, patients may instinctively associate tertiary hospitals with superior care and default to these facilities without fully considering primary care options. In contrast, System 2 engages in analytical reasoning, assessing factors such as treatment adequacy, financial costs, and potential health risks. However, instead of overriding System 1, System 2 often provides post hoc rationalisations that reinforce initial, heuristic-driven choices [4].

This study integrates dual process theory to examine how cognitive mechanisms sustain bypassing behaviour in China’s tiered healthcare system. By differentiating between intuitive and deliberative processes, it provides a deeper understanding of why patients continue to favour tertiary hospitals despite policy efforts to promote primary care utilisation. Specifically, this theoretical framework enables an analysis of:

- Heuristic-driven decision-making (System 1): How automatic cognitive processes, including status quo bias, the availability heuristic, and social reinforcement, contribute to bypassing behaviour.
- Deliberative risk assessment (System 2): How patients consciously evaluate concerns about primary care quality, diagnostic reliability, and treatment effectiveness when making healthcare decisions.
- The interplay between the two systems: How System 2 reasoning rationalises, rather than counteracts, the habitual decisions driven by System 1.

Notably, while a catalogue of specific heuristics is valuable, dual process theory offers the essential overarching framework that explains how such heuristics (System 1) interact with more reflective reasoning (System 2) to produce and entrench behavioural outcomes such as bypassing. This approach moves beyond a mere enumeration of heuristics to model their dynamic role within the cognitive architecture of decision-making.

By applying dual process theory, this study moves beyond existing explanations—primarily structural, perceptual, sociocultural, and economic factors—to uncover the cognitive mechanisms underlying bypassing behaviour. This perspective offers a more nuanced understanding of healthcare decision-making and provides insights for policy and clinical interventions that address both intuitive and deliberative pathways to encourage primary care utilisation more effectively.

### Study Design and Setting

A case study approach was selected for its ability to generate in-depth, context-specific insights into healthcare decision-making [35]. This method is suited to examining cognitive processes, as it enables exploration of both intuitive (System 1) and deliberative (System 2) decision-making within patients’ lived experiences. This study adheres to the COREQ reporting guidelines [28].

The case focuses on hypertensive patients in Shenzhen, China, a population that provides a strategically valuable lens for examining bypassing behaviour within the tiered healthcare system. Hypertension is a chronic condition requiring lifelong, regular monitoring and management, making patients frequent users of both primary and hospital-based services [8]. This high frequency of interaction presents repeated decision-making points where the cognitive processes underlying provider choice can be readily observed. The case selection was driven by two key factors: the high prevalence of hypertension and its intended management at the primary care level. Indeed, hypertension is explicitly designated within Chinese health policy as a condition suitable for management within community health centres [8, 9], making bypassing behaviour a clear deviation from the system’s intended design and a significant policy challenge.

As a rapidly developing urban centre, Shenzhen has witnessed a rising burden of hypertension, which affects approximately 21% of the population. However, awareness (54.34%), treatment (43.48%), and control (25.21%) rates remain suboptimal [22], reflecting ongoing challenges in achieving effective management within the tiered system. These suboptimal outcomes are often linked to fragmented care pathways and patient reliance on overcrowded tertiary hospitals for routine management, a core issue this study seeks to understand. Additionally, Shenzhen has one of the most developed community healthcare infrastructures in China, with nearly 850 community health organisations and approximately 15,000 health professionals as of 2023 [23]. This context provides a rigorous test case; if well-equipped primary care facilities are still being bypassed by hypertensive patients, it suggests that the drivers are not merely infrastructural but are deeply rooted in cognitive and perceptual factors, which is the central focus of this investigation.

### Participants

Participants were purposively selected to ensure the inclusion of individuals with direct experience of bypassing primary care. Eligibility criteria required a formal diagnosis of hypertension and a demonstrated preference for hospital-based care over community health services. Individuals who primarily accessed primary care for hypertension management or had limited autonomy in healthcare decision-making (e.g. those with cognitive impairments) were excluded.

Participants were recruited through outpatient clinics at a tertiary hospital in Shenzhen, using referrals from health professionals familiar to the author. To mitigate potential coercion, these professionals assisted with recruitment solely by identifying potentially eligible patients and providing them with a study information pack. This pack contained a cover letter from the clinic lead introducing the study, which emphasised that participation was voluntary and would not affect their care, a participant information sheet, and the author’s contact details (WeChat and mobile phone number). Interested patients were instructed to contact the author directly. Of the 37 individuals who received information packs in March 2024, 23 subsequently contacted the author and were enrolled in the study (Table 1). The primary reason for non-participation was a lack of response, attributable to a lack of interest or time constraints. This approach facilitated access to individuals actively engaging with higher-tier healthcare facilities. Each participant received RMB 100 upon the interview’s completion as token appreciation for their time.

**Table 1:** Research participants

Characteristics	*n*
Sex (male/female)	15/8
Age, mean (range)	51.04 years (38–61)
Years of hospital service use	
1–5 years	5
6–10 years	13
11–15 years	5
Engagement with community health services	
Never used	17
Occasionally used (e.g. for prescriptions)	6

Data on participants’ educational attainment were not collected. This was a deliberate methodological choice aligned with the study’s aim to explore universal cognitive mechanisms (heuristic and deliberative processes) that underpin health-seeking behaviour. These cognitive processes are not necessarily contingent upon formal education levels but are rather influenced by a complex interplay of lived experience, perceived risk, and system-level factors

### Data Collection and Analysis

Data were collected through semi-structured interviews, lasting 69–83 min, conducted from March to May 2024. Interviews were held in cafés chosen by participants to ensure their comfort and convenience, a strategy aimed at reducing the formality of the setting and fostering more candid discussions than might occur in a clinical environment. To safeguard confidentiality and data quality in this setting, several measures were implemented: (1) Participants were asked to select a quiet time and area within the café to minimise background noise and interruptions; (2) High-quality, directional microphones were used to ensure clear audio recording and minimise the capture of ambient noise; (3) All participants were reassured of confidentiality at the outset, and no personally identifiable information was discussed during the interview; and (4) Transcripts were anonymised during the transcription process, with all names and identifying details replaced with participant codes.

The interview guide, grounded in dual process theory, aimed to explore participants’ health-seeking behaviours, their perceptions of primary care, and the cognitive factors influencing their preference for hospital-based services. Key questions included: ‘Can you describe a typical visit to the hospital for your hypertension care? What influences your decision to choose the hospital over a community health centre?’, ‘Have you considered the benefits or risks of using primary care for hypertension management? What factors do you consider when making this decision?’, and ‘Do you think your past experiences with hospital services influence your decision to go to the hospital even when primary care might be available? Why do you think that is?’

The interviews were audio-recorded, transcribed verbatim, and the transcripts were analysed in Chinese to preserve the original context and meaning. A deductive thematic analysis was conducted, guided by the theoretical framework. The analysis focused on three key themes: the role of System 1, where heuristic-driven decision-making, shaped by status quo bias, the availability heuristic, and social reinforcement, sustained bypassing behaviour (e.g. habitual hospital preference influenced by past experiences and social norms); the influence of System 2, involving reflective deliberation and risk assessment related to treatment adequacy, misdiagnosis concerns, and perceptions of primary care quality; and the interplay between the two cognitive systems, where System 2 justifications reinforced, rather than countered, System 1 biases, sustaining the default preference for higher-tier facilities.

The analysis was performed using ATLAS.ti, with all interview transcripts independently coded by the author, who has extensive experience in community health services research. The coding process followed a deductive approach, with initial codes derived from the theoretical framework and interview questions. Each transcript was thoroughly reviewed, and relevant excerpts were assigned codes reflecting key concepts related to the cognitive mechanisms underpinning patients’ healthcare decisions. The coding process was iterative, with codes refined and reorganised as themes developed. Consistency checks were regularly conducted by reviewing the coded data across all transcripts to ensure the reliability and validity of the analysis. Thematic refinement was an ongoing process, ensuring depth and clarity. Member-checking was carried out with two randomly selected participants to enhance the accuracy and credibility of the findings. Data saturation was reached after 23 interviews, as no new themes emerged from the final interviews.

To ensure the credibility and trustworthiness of the analysis despite employing a single coder, a multi-step process was implemented. First, a comprehensive audit trail was maintained within ATLAS.ti, meticulously documenting all coding decisions, analytical memos, and the evolution of the codebook. Second, an iterative process of re-examination was undertaken whereby codes and emerging themes were consistently reviewed against the raw data to ensure coherence and fit. Third, to address potential interpretive bias, negative case analysis was actively pursued; instances that contradicted the developing patterns, such as participants expressing a preference for primary care, were explicitly sought and integrated into the analysis, culminating in the theme covering divergent cases. Finally, a form of peer debriefing was conducted through discussions with academic colleagues not involved in the research. These sessions were used to challenge the coding framework, thematic structure, and the interpretation of representative excerpts, thereby testing the logical soundness of the findings.

### Reflexivity

The author is a male postdoctoral fellow with a disciplinary background in the sociology of public health and extensive experience as a qualitative researcher specialising in community healthcare systems.

No participants were acquainted with the author prior to their involvement in the study. They were informed that the research was being conducted by an academic from The Hong Kong Polytechnic University seeking to understand how patients make decisions about their healthcare. The broader research interest in patient decision-making was disclosed, though the specific theoretical framework (dual process theory) was not introduced to avoid predisposing participants to its concepts and to prevent potential confusion or discomfort with theoretical terminology, thereby ensuring responses remained grounded in their personal experiences.

The author’s professional background in community health research inevitably informs his interpretation of the data, generating a strong awareness of the structural and policy challenges facing primary care. This carried an inherent risk of a priori bias, specifically a tendency to frame bypassing behaviour as a problem to be solved rather than a phenomenon to be understood. Consequently, a conscious practice of reflexivity was maintained throughout the analysis. This involved deliberately suspising judgement during coding and repeatedly returning to the raw transcripts to ensure that the emerging analysis remained grounded in participants’ own rationalisations. The goal was to guard against a deficit-based interpretation of patient choices and to remain genuinely open to the internal logic of their reasoning.

This commitment to understanding the patient’s perspective, rather than evaluating it, made dual process theory a suitable analytical framework. It offered a structured lens through which to neutrally analyse the reasoning behind bypassing behaviour, thereby helping to mitigate potential bias arising from the author’s professional advocacy for strengthened primary care. The deductive application of this theory, supplemented by an openness to emergent themes, facilitated an analysis that is both systematically rigorous and firmly grounded in participants’ lived experiences.

### Ethics

Informed Consent was obtained verbally by the author prior to the commencement of each interview. The process involved a detailed explanation of the study’s purpose, procedures, and the strict measures in place to ensure confidentiality. Participants were explicitly informed of their right to decline to answer any question, to pause, or to withdraw from the study at any time without any consequence to their medical care. This process was conducted in Mandarin to ensure full comprehension. Verbal consent was deemed most appropriate and was approved by the ethics committee to avoid creating a paper trail containing personal identifiers, thus further protecting participant anonymity. Oral consent was audio-recorded as part of the interview file to maintain a verifiable record. Confidentiality was maintained through data anonymisation and secure storage, with all identifiable information removed and quotations attributed using pseudonymous codes (IW1–IW23). This study was ethically approved by The Hong Kong Polytechnic University Institutional Review Board, adhering to established ethics standards.

## Results

### Thematic Development

An initial codebook was developed based on dual process theory. A priori codes were established to capture System 1 processes (e.g. automatic habit, familiarity bias, social norm influence, anecdotal reasoning) and System 2 processes (e.g. risk assessment, cost benefit analysis, comparative evaluation, long-term planning).

The analysis was iterative, with codes being continuously refined and expanded. For instance, the code ‘automatic habit’ was later specified into more precise child codes such as ‘status quo bias routine’ (e.g. ‘I’ve been going to the hospital for years’) and ‘status quo bias avoidance’ (e.g. ‘Why change something that works?’), directly reflecting participants’ attachment to established patterns of care-seeking. Similarly, instances of ‘anecdotal reasoning’ were found to align closely with the availability heuristic. This inductive insight led to its formalisation into the more precise code ‘availability heuristic negative’ to capture vivid, easily recalled stories, whether personal (e.g. unsatisfactory clinic visit) or social (e.g. friend’s delayed diagnosis), that disproportionately influenced perceptions of primary care risk.

The code ‘social norm influence’ was applied to narratives involving family or peer pressure. During second-cycle coding, it became clear that this influence often functioned as ongoing reinforcement rather than one-time advice. This inductive refinement process led to the theme ‘social reinforcement’ (e.g. family assertions that ‘real doctors’ are found in hospitals).

Within System 2, finer distinctions emerged between types of reflective deliberation. Codes such as ‘risk diagnostic uncertainty’ and ‘risk treatment adequacy’ were grouped to form the theme ‘conscious assessment of clinical risk’. Deliberations concerning chronic disease management (e.g. comparison of monitoring practices) were captured under ‘long-term management evaluation’, leading to the theme ‘deliberative consideration of long-term management’.

The most significant analytical insight was the consistent interaction between Systems 1 and 2. A pattern was observed in which System 2 codes (e.g. explicit risk assessment) were used to rationalise, rather than reevaluate, System 1 impulses (e.g. status quo bias). This led to the overarching theme ‘the interplay of cognitive systems’, with sub-themes addressing rationalisation and reinforcement.

Finally, divergent cases were identified through the application of the theoretical codes, where utilitarian codes such as ‘convenience’ or ‘accessibility’ outweighed ‘perceived risk’ or ‘social influence’, illustrating exceptions to the dominant bypassing behaviour.

### System 1: Heuristic-Driven Bypassing

Participants’ decisions to bypass primary care were predominantly shaped by heuristic-driven reasoning, reinforcing a habitual preference for hospitals. Three key cognitive mechanisms emerged: status quo bias, availability heuristic, and social reinforcement.

#### Status Quo Bias

Status quo bias was evident in participants’ strong inclination to seek hospital care out of familiarity. Many described their reliance on hospitals as an unquestioned routine rather than a consciously evaluated choice. IW4 (53, male) stated, ‘I’ve been going to the hospital for years. It never occurred to me to try the community clinic. Why change something that has always worked for me?’ IW11 (56, male) echoed this sentiment: ‘I don’t know much about the community clinic, but I do know the hospital. I trust what I’m familiar with.’

#### Availability Heuristic

The availability heuristic reinforced hospital preference, as participants based their perceptions of primary care on easily recalled experiences, often negative ones. Past encounters, either personal or from acquaintances, shaped an enduring distrust of primary care’s reliability. IW9 (47, male) recounted, ‘I went to the community clinic once, and they just gave me some medicine without really checking my condition. I had to go to the hospital later anyway. Since then, I don’t bother with the clinic.’ IW15 (50, male) described how second-hand experiences influenced their perception: ‘A friend went to the clinic for a check-up and was told everything was fine. A few weeks later, he had to rush to the hospital because his blood pressure shot up. Stories like that make me think it’s not worth the risk.’ These isolated incidents, though not necessarily representative, disproportionately shaped participants’ risk assessments, fostering a belief that hospital care was the safer option.

Participants’ descriptions of hospital visits further underscored the ingrained nature of their preference. Many recounted their visits as structured and predictable, reinforcing a sense of reassurance. IW2 (51, female) explained, ‘I take the bus early in the morning to get a queue number. Then I see the doctor, get my blood pressure checked, and pick up my medication. It takes a few hours, but at least I know I’m getting proper care.’ IW14 (49, female) emphasised perceived differences in expertise: ‘Hospital doctors are specialists. They see complicated cases every day, so they know what they’re doing. Community doctors just deal with minor issues.’ Even when acknowledging long waiting times, participants viewed hospital care as a necessary trade-off: ‘Yes, I have to wait, but it’s worth it. I’d rather wait longer and see a real doctor than take a risk with a clinic where they might not catch something important’ (IW10; 55, male).

#### Social Reinforcement

Social reinforcement sustained hospital preference. Many participants reported that their family, friends, or colleagues viewed hospital care as the superior option, shaping their own attitudes. IW6 (60, female) explained, ‘My family always tells me to go straight to the hospital. They say, “If you want real doctors, you don’t go to the community clinic.” So that’s what I do.’ IW20 (54, male) noted, ‘Everyone I know goes to the hospital. If primary care were good enough, people wouldn’t still be lining up at hospitals. That says a lot.’

### System 2: Deliberative Decision-Making

System 2 decision-making involves reflective, deliberate thought processes activated when patients assess the risks and benefits of healthcare options. Unlike the automatic, heuristic-driven decisions of System 1, System 2 is characterised by active, effortful evaluations of treatment adequacy, diagnostic reliability, and the perceived quality of care. When deciding between primary care and hospital services, patients engage in this reflective process, weighing the potential risks associated with each choice.

#### Conscious Assessment of Clinical Risk

Participants described evaluating the risks of primary care, with particular emphasis on deliberating concerns regarding the adequacy of treatment and the reliability of diagnoses in community health centres. IW12 (47, male) explained their reasoning process: ‘I know the community health centres might not have the same level of experience with hypertension, and there are fewer diagnostic tools available. I worry and calculate that if they miss something, it could make my condition worse. After thinking it through, I just go to the hospital because I conclude it’s more secure.’ Similarly, IW2 reflected on a comparative assessment: ‘At the hospital, they have all the tests and equipment to properly diagnose my condition. I’ve assessed that I don’t trust the smaller clinics to catch everything, especially when it comes to something serious like hypertension.’

This conscious risk assessment extended to concerns about treatment quality. IW1 (55, male) articulated a calculated decision: ‘I’m not sure the community health centre would be able to provide the right kind of treatment for me. I’ve considered that if my hypertension gets worse, I’d need more specialised care, and I’ve therefore decided to trust the hospital to handle that better.’

#### Deliberative Consideration of Long-Term Management

Additional to immediate risks, participants described engaging in deliberate thought about the long-term implications of relying on primary care for managing hypertension. IW9 explained their longitudinal analysis: ‘I’ve been to the community health centre before, but I’ve concluded that they don’t seem to pay as much attention to the long-term management of my condition. I’ve compared this to the hospital, they monitor me more closely, and I therefore feel like they have a better understanding of my health history.’

The perceived expertise of hospital staff was also a product of conscious comparison rather than mere instinct. IW9 elaborated on this judgement: ‘When I go to the hospital, I reason that I’m in good hands because the doctors are specialists. I base this on the knowledge that they’ve been trained specifically for hypertension and I judge that they understand it better than the general doctors at the community health centre.’

### The Interplay of Cognitive Systems: Reinforcing Bypassing Behaviour

The decision to bypass primary care and seek hospital services is shaped not only by System 1’s automatic, heuristic-driven processes but also by the reflective evaluations of System 2. Critically, the interaction is synergistic rather than competitive; System 2 reasoning seeks out justifications that reinforce, rather than counterbalance, System 1’s initial preferences, leading to a sustained reliance on higher-tier healthcare.

#### Rationalising the Intuitive Choice

Participants described a process where an initial, intuitive preference for hospitals—an automatic feeling based on past experiences or a sense of security (System 1)—was subsequently validated through conscious, deliberate reasoning (System 2). IW8’s (57, female) account illustrates this sequence: ‘I always go to the hospital; it just feels more reliable.’ [System 1 intuition]. ‘I know it’s more expensive, but I don’t want to take any chances. When I think about it, I feel it’s the safest option, and I trust it more.’ [System 2 justification]. This reflects how System 2 engages in motivated reasoning, rationalising the heuristic-driven choice in terms of reliability and safety.

Despite acknowledging primary care’s potential benefits abstractly, participants described how their automatic preference was bolstered by deliberate reasoning. IW5 (58, male) noted: ‘I know primary care might be good for minor issues,’ [System 2 acknowledgement of alternatives] ‘but for something like hypertension, I can’t just rely on them. I think about it, and I feel the hospital is better equipped. They have all the tests and equipment.’ [System 2 justification reinforcing System 1 choice].

This process of seeking justifications involved a conscious weighing of risks, which invariably favoured the hospital. IW15 stated: ‘The clinic is closer and cheaper, but when I think about my condition long-term, I wonder if they can handle it.’ [System 2 risk assessment]. ‘I always go to the hospital because they have specialists who really understand hypertension.’ [System 2 justification]. IW14’s account provides a further example of this deliberative reinforcement: ‘I’ve been seeing doctors at the community health centre for a while, but I always think: what if they miss something? What if I end up with a stroke?’ [System 2 risk assessment] ‘At the hospital, they have more experience with serious conditions like mine. I’ve had several tests there, and I feel more secure about my treatment.’ [System 2 justification].

#### Reinforcement Through Negative Experiences

Some participants reflected on how a specific, salient negative experience (processed initially by both systems) created a powerful feedback loop that solidified the bias. IW7 (51, female) recalled: ‘The first time I went to the community health centre, I felt like they didn’t take my hypertension seriously. It wasn’t like the hospital, where they checked everything.’ [Initial experience creating a strong System 1 association & System 2 evaluation]. ‘That experience made me trust hospitals more. Now, I don’t even think twice about it.’ [The resulting System 1 habit entrenched].

While participants’ reflective evaluations provided rationales for bypassing primary care, these evaluations functioned primarily to reinforce, rather than to critically challenge, their initial biases. As IW3 (55, female) concluded: ‘I’m aware the community health centre might be fine for some people,’ [System 2 superficial acknowledgement] ‘but for me, I prefer the hospital. I’ve thought about it, and I just feel safer there.’ [System 2 justification reinforcing System 1 intuition].

### Divergent Cases: A More Balanced Perspective

While most participants exhibited a strong preference for hospital services, a few cases revealed a more balanced perspective, with participants weighing the merits of both primary care and hospital services. They demonstrated less pronounced biases towards hospital care, influenced by their health status and personal circumstances.

Several participants were younger, had less severe hypertension, or were free from additional health complications, which allowed them to consider primary care as a viable option. For example, IW16, a 39-year-old male with mild hypertension, noted: ‘I don’t mind going to the community health centre sometimes. I think it’s just as good for me right now. I get regular check-ups and my condition is under control, so I don’t feel the need to go to the hospital all the time.’

Additionally, some participants with demanding work schedules showed greater flexibility in their healthcare choices. IW13, a 44-year-old male professional with mild hypertension, explained: ‘I don’t always have time for the hospital. The clinic is much more convenient, especially since they don’t require long waits. I’ve been there a few times, and I trust them for routine management.’

These participants were less likely to view hospital services as essential, instead considering primary care a legitimate alternative. Their decisions were shaped by less entrenched System 1 biases, with System 2 deliberation resulting in a more neutral, or even favourable, evaluation of primary care. As IW13 further stated: ‘I used to think the hospital was always the best choice, but now I feel comfortable with the community health centre. They know me, and it’s easier to get in for a quick check-up.’

## Discussion

This study examines the cognitive mechanisms underpinning primary care bypassing among hypertensive patients in a Chinese city through the lens of dual process theory. Findings highlight how heuristic-driven System 1 processes reinforce habitual hospital use, while deliberative System 2 reasoning often serves to justify, rather than counter, these automatic preferences. Nevertheless, some participants, shaped by specific demographic and contextual factors, adopt a more balanced approach to healthcare decision-making, suggesting that bypassing behaviour is not wholly determined by cognitive biases.

Consistent with dual process theory [3, 4], System 1 thinking emerges as a central driver of bypassing behaviour. Participants demonstrate status quo bias, maintaining established hospital routines despite the availability of primary care services. The availability heuristic further entrenches this pattern, as prior experiences of hospital-based care, coupled with anecdotal accounts from family and peers, reinforce the belief that hospitals are the only reliable option. Such cognitive shortcuts are well-documented in healthcare literature, where habitual decision-making fosters an overreliance on specialist care, even for chronic conditions that could be effectively managed in primary care settings [2, 16, 26].

Social reinforcement plays a critical role in shaping healthcare decisions. Many participants report that family members and social circles overwhelmingly favour hospital care, further entrenching their preference for hospital-based treatment. This aligns with existing research that underscores the influence of collective beliefs and cultural narratives in shaping health-seeking behaviours [7, 14, 29, 36]. The findings suggest that healthcare decision-making is not solely an individual cognitive process but is strongly shaped by the social environment and the broader cultural context in which individuals operate [29].

Importantly, System 2 reasoning, typically associated with reflective deliberation, often works in tandem with System 1 heuristics, reinforcing the default preference for hospital care. Although participants acknowledge the potential benefits of primary care, such as convenience and lower costs [25], their rationalisations for hospital visits are consistently underpinned by uncertainties about the adequacy and reliability of community-based services [10, 34]. These perceived inadequacies [37], as previously outlined, are often reflections of very real structural and economic disparities, such as resource gaps and financial disincentives for primary care providers. This suggests that bypassing behaviour is not simply the result of a lack of information or awareness [31] but is rooted in deeply ingrained cognitive biases and risk perceptions that are often reinforced by the existing healthcare system structure.

These biases are further reinforced by a hierarchy of healthcare quality, where hospital care is regarded as inherently superior [10, 16, 34]. For many participants, justifications for choosing hospital services are often not based on a rational evaluation of healthcare options, but rather on a default reliance on familiar, higher-tier facilities perceived as safer and more capable of managing complex medical conditions [16]. This cognitive reinforcement loop indicates that bypassing behaviour is a multifaceted issue, involving not only ‘misperceptions’ of primary care’s capabilities [34, 37] but also broader habitual thought patterns and entrenched societal norms that prioritise hospital care over primary care [27, 29].

While most participants exhibit a strong preference for hospital care, a subset of individuals demonstrate a more balanced decision-making approach, indicating that bypassing behaviour is not entirely deterministic. These individuals—typically younger, busier, or with less severe conditions—are more likely to utilise primary care when it offers greater convenience or shorter waiting times. Their less entrenched reliance on past experiences and a lower perceived risk of serious complications appear to foster more flexible, context-dependent decision-making. This finding challenges the assumption that cognitive biases are an ‘all-encompassing’ driver of health-seeking behaviour [1]. It suggests that contextual factors, such as age, the severity of the condition, and external pressures like time constraints, can mitigate the impact of habitual biases, facilitating a shift towards primary care utilisation. However, it is crucial to recognise that even in these cases, the underlying influence of cognitive heuristics and social norms still plays a role in shaping their decisions, albeit to a lesser extent. This underscores the need for a nuanced understanding of healthcare decision-making, one that considers not only cognitive processes but also individual variability and situational contexts.

Collectively, these findings help to delineate the boundary conditions of the dual-process model of bypassing behaviour. The self-reinforcing cycle of heuristic-driven choice and deliberative justification appears most dominant among older patients managing more severe chronic conditions, and those within strong social networks that normalise hospital use. The model’s predictive strength is therefore greatest in contexts where perceived risk is high and structural alternatives are viewed as weak. Conversely, the effect is attenuated, though not negated, by factors that reduce the cognitive and social stakes of the decision. These include younger age, less severe illness, time constraints that elevate the value of convenience, or weaker social reinforcement for institutional care. These boundaries clarify that whilst dual-process mechanisms are fundamental, their behavioural expression is moderated by individual differences and situational contexts. This delineation not only sharpens the study’s theoretical precision but also provides a more nuanced framework for designing targeted interventions aimed at specific patient profiles.

### Limitations

This study has several limitations. First, the deductive approach may have constrained the analysis by focusing attention on pre-defined cognitive processes, potentially overlooking other salient, emergent themes or alternative explanations for bypassing that fall outside the theoretical framework. For instance, the role of unmeasured emotional factors (e.g. anxiety) or more nuanced structural barriers not directly linked to cognition may be underexplored.

Second, the focus on hypertensive patients limits generalisability. Specifically, the nature of hypertension (a chronic, often asymptomatic condition that carries a significant risk of severe complications) may bias patient preferences towards hospital care. This population may be particularly risk-averse and more likely to seek perceived ‘safer’, specialist-led care in hospitals, potentially amplifying the bypassing behaviour under investigation. Consequently, their healthcare decisions may not reflect those of individuals with acute, self-limiting, or highly symptomatic conditions, who might prioritise convenience over perceived safety, or those with multi-morbidities whose care pathways are more complex.

Third, while the inclusion of divergent cases is a strength of qualitative research for providing a nuanced understanding of the phenomenon, it also delineates a boundary of the findings. The perspectives of younger patients or those with less severe hypertension, who exhibited more flexible decision-making, are essential for a complete analytical picture but may not be representative of the broader hypertensive population who more rigidly bypass primary care. This variation underscores that the cognitive mechanisms identified are influential but not deterministic, and their salience is likely moderated by individual factors such as disease severity and age.

A further limitation is that this study did not examine the potential moderating effect of socio-economic status, including educational attainment. Consequently, the analysis may not capture how these fundamental cognitive processes are nuanced by social stratification.

Additionally, the study is limited to Shenzhen, an urban setting with a well-developed community healthcare system, and findings may not fully capture bypassing behaviours in other contexts with weaker primary care infrastructure, where the calculus of choice may be dominated by absolute availability of services rather than perceptions of quality.

Finally, the recruitment of participants actively using hospital services, while necessary for the research, excludes the perspectives of those who do not bypass care. Their insights could have provided a valuable counterpoint for understanding the factors that enable successful primary care engagement.

### Implications for Policy and Clinical Interventions

Addressing the cognitive and social drivers of bypassing behaviour requires multidimensional interventions that reshape patient perceptions and decision-making. Strengthening trust in primary care can be achieved through regular specialist consultations at community health centres, allowing patients to access expert opinions without defaulting to hospitals. Shenzhen’s current practice of hospital specialists stepping down to community clinics already supports this approach but could be expanded and institutionalised to bridge the gap between primary and tertiary care more effectively.

Enhancing physician continuity in primary care is also critical. Sustained patient-provider relationships can help mitigate concerns about diagnostic reliability, fostering familiarity and trust in community-based services. To further encourage first-contact visits, system-level changes such as default primary care registration should be introduced, nudging patients towards community health centres rather than bypassing them for hospital services.

Financial interventions are also crucial in shaping healthcare choices. Shenzhen’s tiered insurance system offers an opportunity to incentivise primary care use. Local residents with tier-1 insurance can freely choose healthcare providers, suggesting that financial measures, such as reduced co-pays or higher reimbursements for primary care visits, could encourage greater utilisation of community health centres. For most non-local residents, whose tier-2 insurance is tied to specific community health centres for reimbursement, optimising reimbursement structures and ensuring equitable access to quality care could help reduce bypassing.

Moreover, leveraging social networks is essential in shifting entrenched healthcare-seeking norms. Community health advocates and family physicians can serve as trusted figures to promote primary care, challenging the prevailing perception that hospitals are the superior choice.

## Conclusion

This study applies dual process theory to explore the cognitive mechanisms behind primary care bypassing among hypertensive patients in Shenzhen, China. Findings demonstrate that System 1 heuristics, reinforced by habitual hospital use and social norms, establish bypassing as the default behaviour, while System 2 reasoning typically justifies rather than challenges these preferences. Although some patients, influenced by contextual factors such as health status and convenience, exhibit more flexible decision-making, the broader pattern suggests that bypassing is rooted in both cognitive and social factors. The study offers a novel account of the interplay of cognitive processes in healthcare decision-making within China’s tiered healthcare system. By applying dual process theory, it enhances understanding of how automatic and deliberative thinking influence bypassing behaviour. Additionally, it underscores the need to consider both individual cognitive biases and societal norms when developing interventions aimed at improving primary care utilisation.

## Data Availability

Data are available from the author upon request.
